# A Case Report of Revision Total Knee Arthroplasty After 17 Years: All Grown Up, What Happens When Implants Mature?

**DOI:** 10.7759/cureus.2797

**Published:** 2018-06-13

**Authors:** Colin K Cantrell, Harshadkumar A Patel, Wesley R Stroud, Nicholas Dahlgren, Eva Lehtonen, Morad Qarmali, Kelly C Stéfani, Ashish Shah, Sameer M Naranje

**Affiliations:** 1 UAB School of Medicine, University of Alabama at Birmingham, Birmingham, USA; 2 Orthopaedic Surgery, University of Alabama at Birmingham, Birmingham, USA; 3 Orthopaedic Surgery, University of Alabama at Birmingham, Mountain Brook, USA; 4 School of Medicine, University of Alabama at Birmingham, Birmingham, USA; 5 Miller School of Medicine, University of Miami, Miami, USA; 6 Pathology, University of Alabama at Birmingham, Birmingham, USA; 7 Orthopaedic Surgery, Hospital Do Servidor Público Estadual, Sao Paulo, BRA

**Keywords:** revision tka, loosening, follow-up, case report, total knee arthroplasty

## Abstract

The number of total knee arthroplasties (TKAs) being performed annually is steadily rising. Recommendations for clinical follow-up guidelines following these arthroplasties is controversial, with no strict guidelines for long-term follow up. Although a few case series exist which identify a minority of patients who require revision TKA for aseptic loosening or pain more than 15 years after index surgery, no published studies have yet described these patients or the pathology present at the time of surgery in detail. We present the case of a patient who underwent revision TKA for pain and instability that developed 17 years after index surgery. Postoperative pathology revealed foreign body giant cell reaction of the tissue surrounding the previous implant. This case of revision after more than 17 years attempts to improve our understanding of long-term reactions to implants and highlights the necessity of long-term follow up in patients with TKA. It is one of the longest follow-ups of TKA reporting long-term anatomic changes at the bone cement interphase and around the implant.

## Introduction

For more than a decade, the number of total knee arthroplasties (TKAs), primary and revision, has been steadily increasing [1]. With approximately 8% of them being revision TKAs, the documented reasons and timing for these revisions vary greatly [1-2]. Clinical follow up after TKA is controversial, and with the American Association of Hip and Knee Surgeon’s (AAHKS) current recommendation of follow up annually or biennially with no definitive end date, adherence to these guidelines is questionable [3-4]. The longest documented follow up ranges up to 25 years, although complications may occur past this point [5]. One such complication, as presented in this case, is foreign body giant cell reaction [6].

## Case presentation

An 83-year-old female with bilateral primary TKA performed 17 years prior presented to the clinic. The patient was referred with worsening left knee pain, reported gait instability, and swelling for three months duration. Until this point, she had been completely asymptomatic. She was initially seen and treated by an orthopaedic surgeon from an outside facility with physical therapy, followed by a left knee arthrocentesis to rule out infection. The aspirate demonstrated proteinaceous fluid with few benign inflammatory and epithelial cells and cultures were found to be negative. Due to the increasing pain, gait instability, and discomfort, coupled with lack of relief by the current measures, she was referred to the orthopaedic surgery clinic at our institution for further evaluation. 

**Figure 1:**
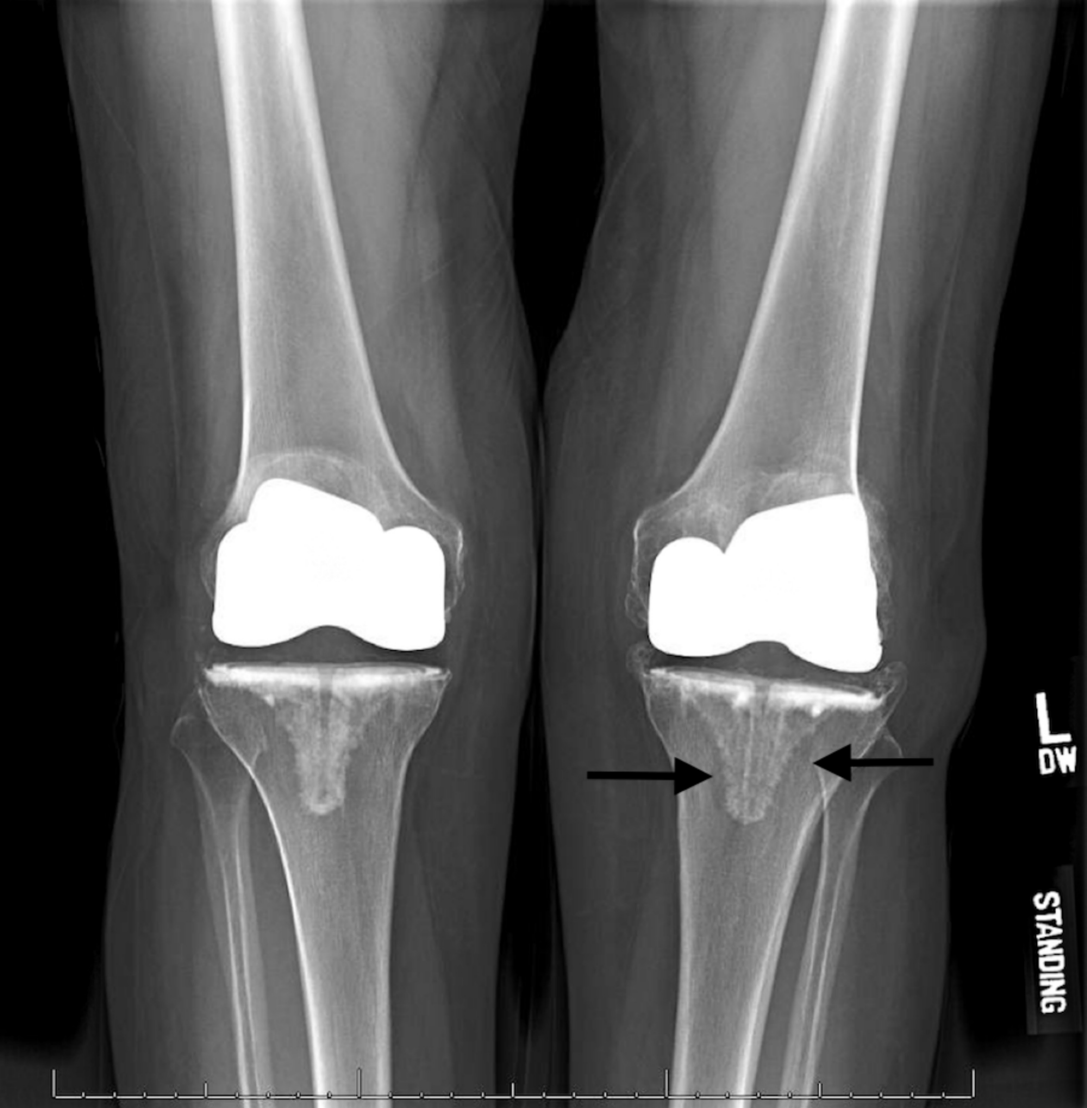
Standing radiograph of the case at initial presentation showing lysis and subsidence of left tibial component

**Figure 2:**
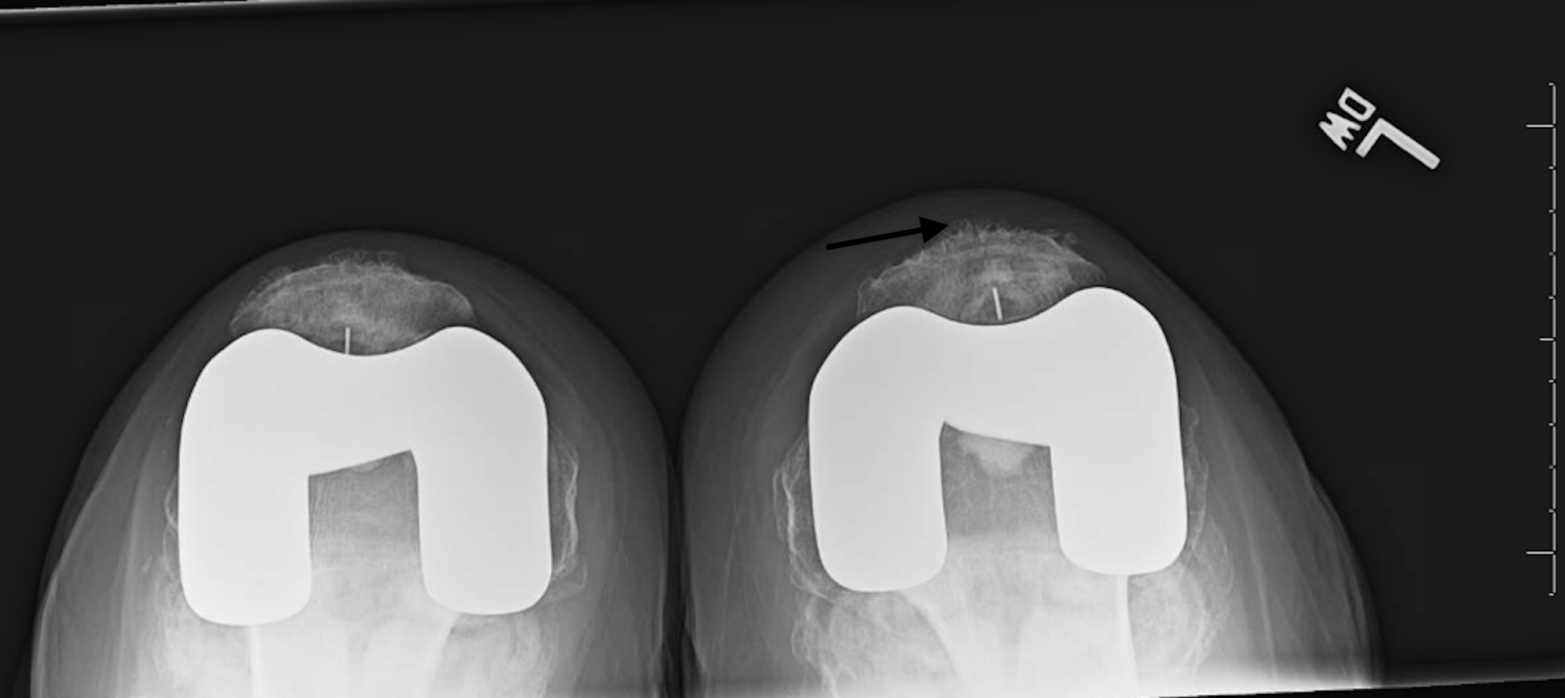
Radiograph of the case at initial presentation showing osteochondral bodies in left suprapatellar recess

**Figure 3:**
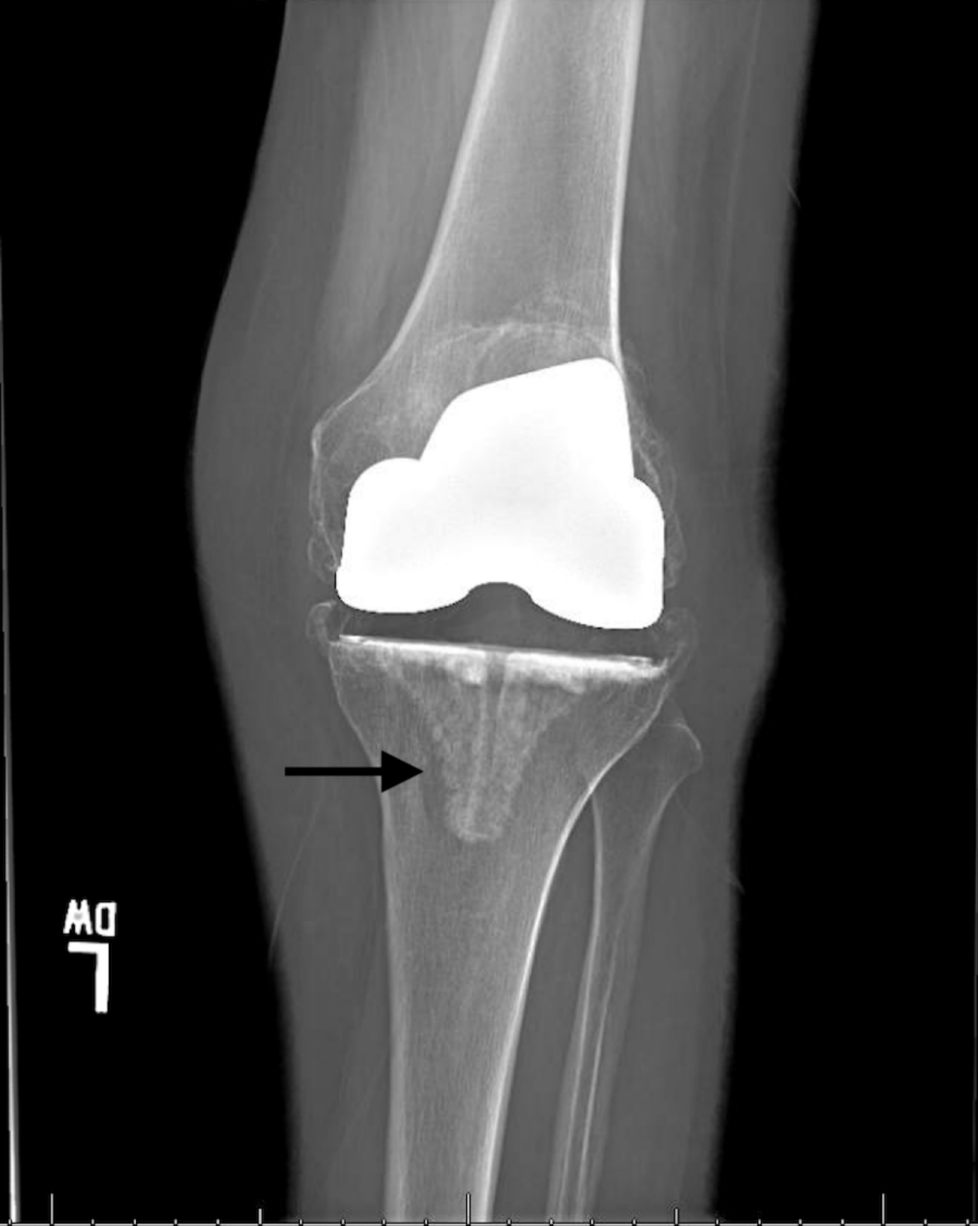
Anteroposterior radiograph of left knee at presentation showing lysis and subsidence of tibial component

**Figure 4:**
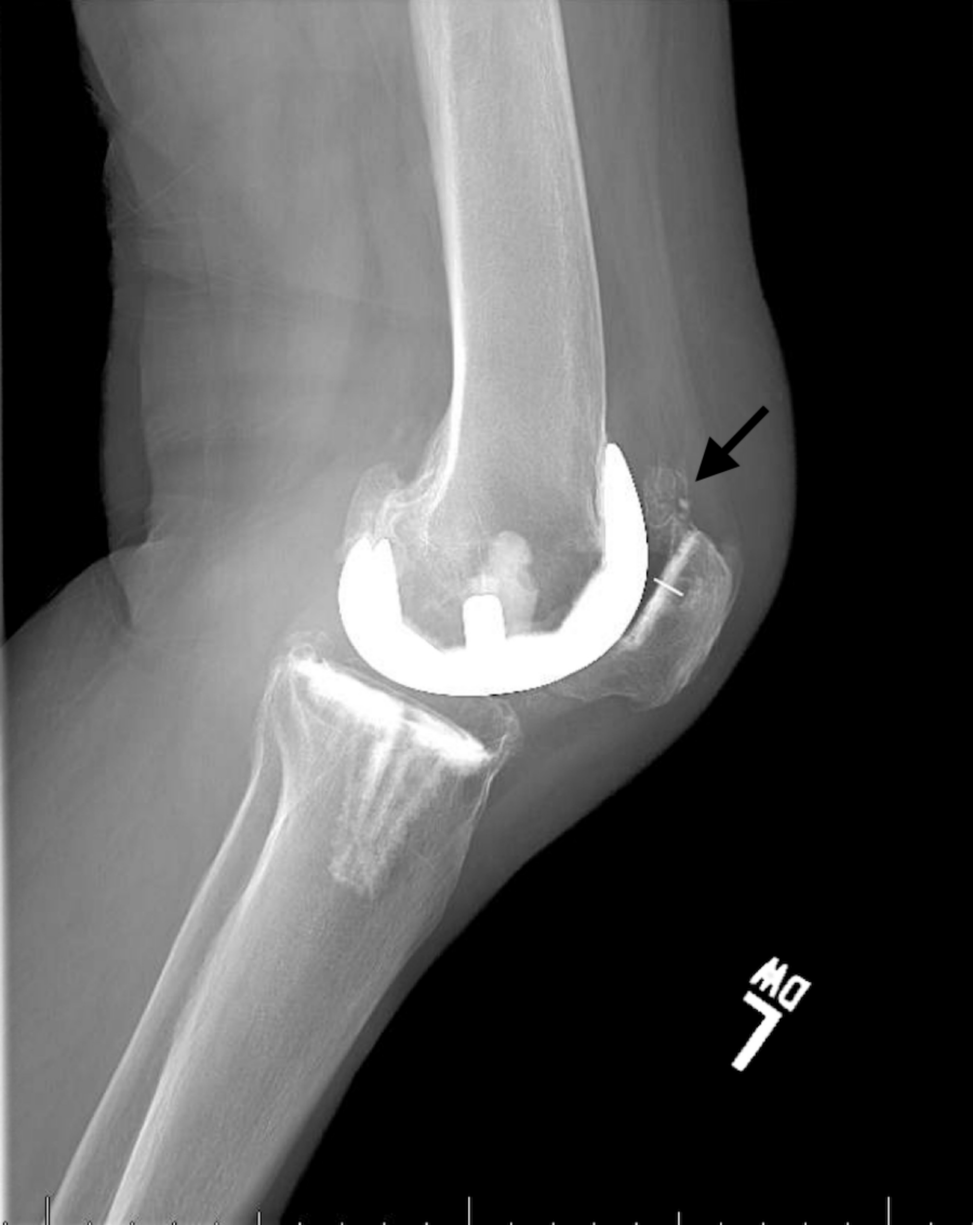
Lateral radiograph of left knee at presentation showing large osteochondral bodies in suprapatellar recess

At her initial visit, the patient reported steadily increasing, sharp pain localized to her left knee joint with associated swelling that worsened with ambulation and prolonged standing and lacked improvement with conservative management. Her day-to-day activities were becoming restricted secondary to the pain and she reported occasional falls due to the perceived instability of her knee joint. Physical exam revealed a mild antalgic gait and tenderness over her proximal tibia. An in-house X-ray was notable for an increase in size and number of osteochondral bodies in the left suprapatellar recess with a left joint effusion and “lysis and subsidence of the tibial component and decreased thickness, suggestive of loosening and wear” (Figures 1-4). The patient then underwent a bilateral knee bone scan which confirmed the tentative diagnosis of implant loosening with polyethylene wear and instability. The patient was counseled on her treatment options, including surgical and non-surgical management, and elected to undergo revision surgery of her left knee arthroplasty.

**Figure 5:**
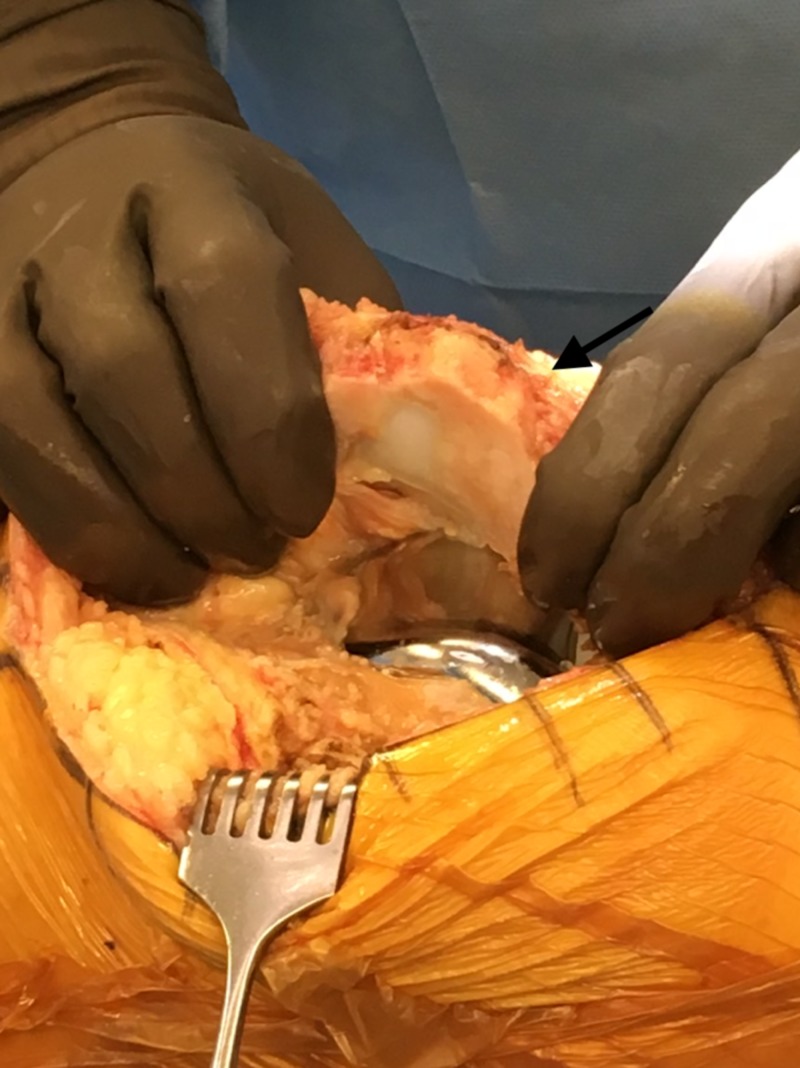
Intraoperative image of patella upon exposure showing extensive osteophyte development

**Figure 6:**
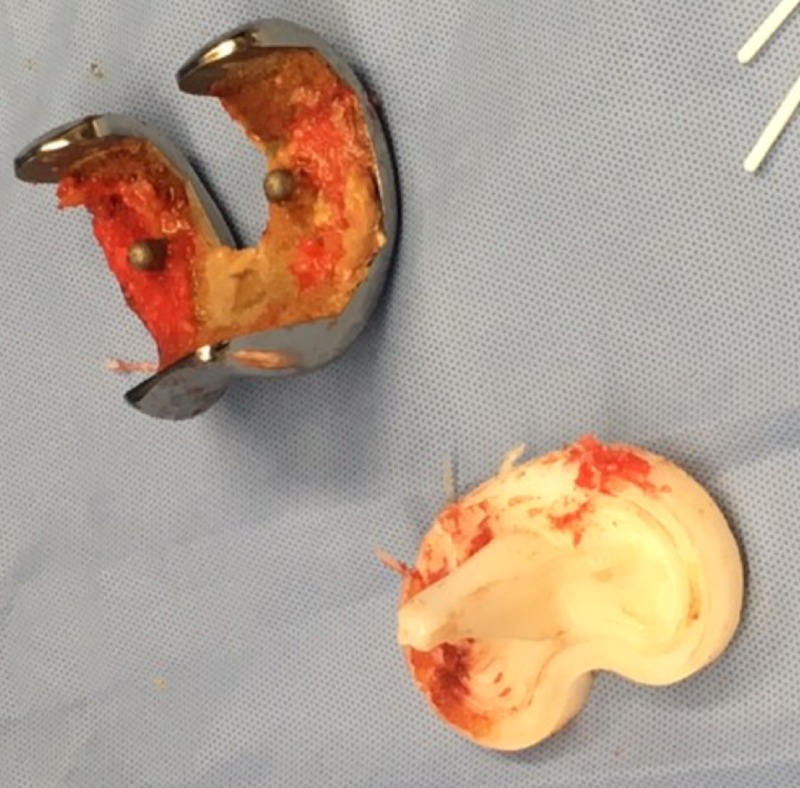
Intraoperative image showing removal of previous total knee arthroplasty (TKA) components

**Figure 7:**
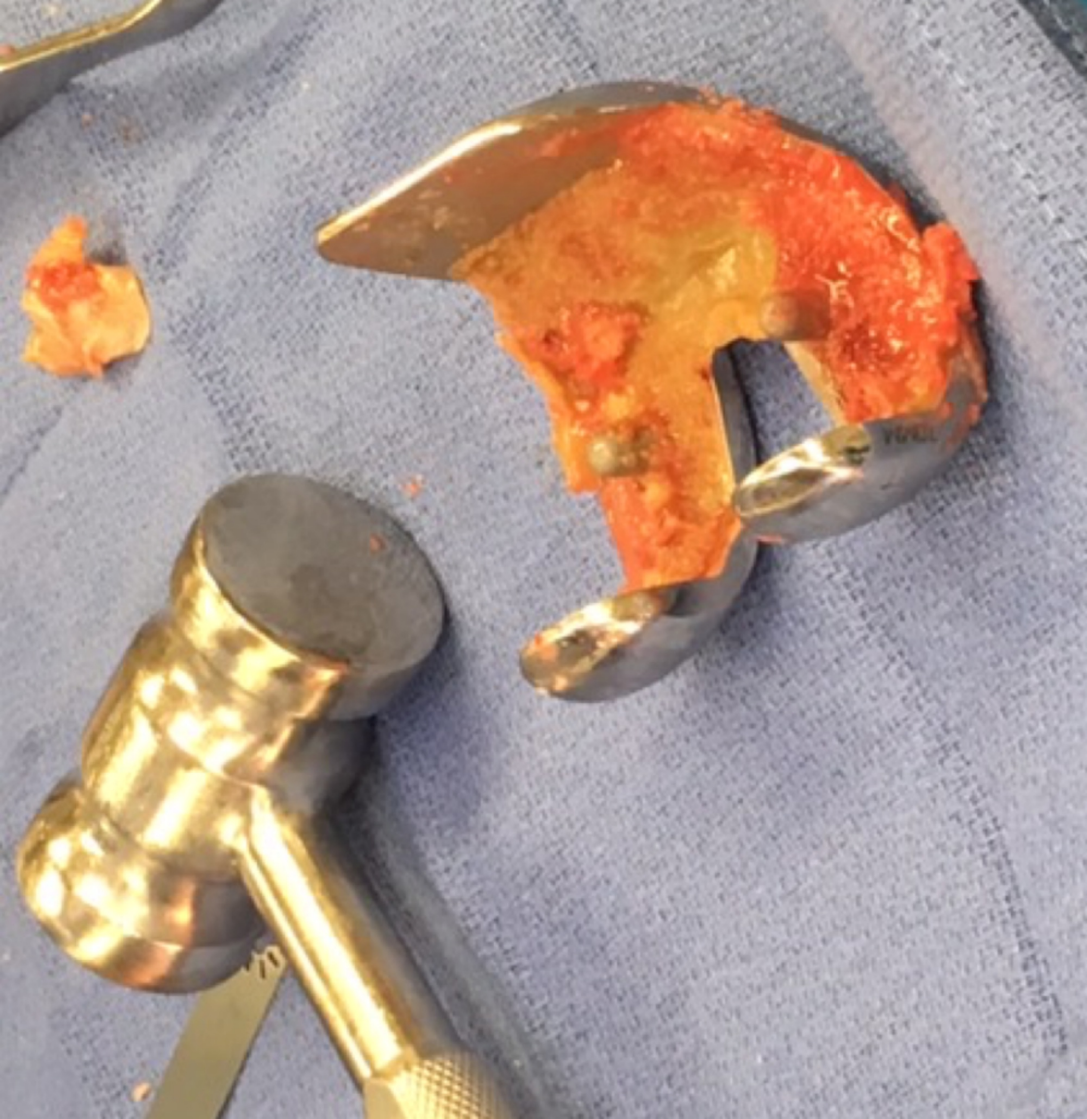
Intraoperative image showing bone ingrowth on femoral component of the previous implant

**Figure 8:**
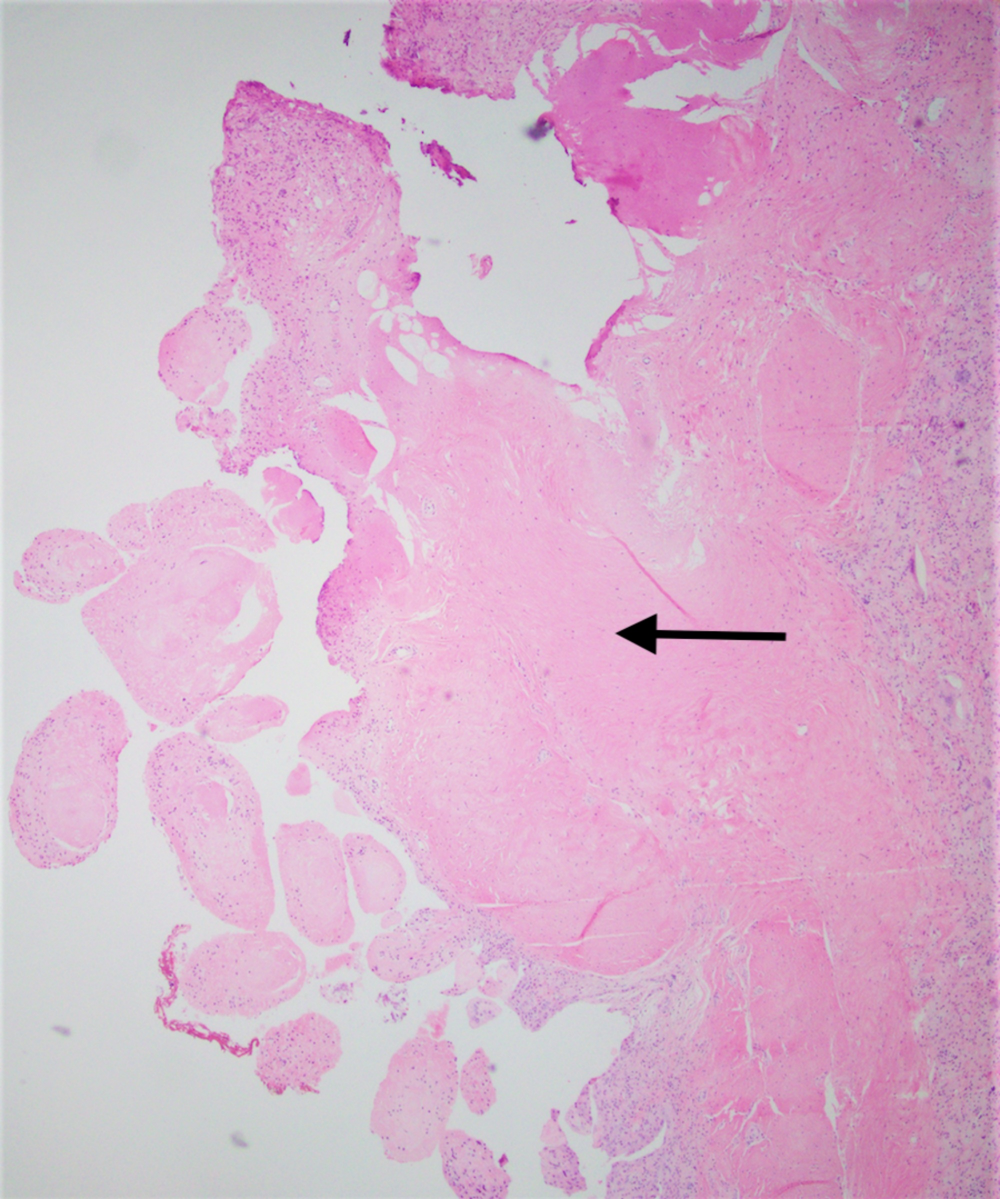
Low power photomicrograph showing papillary synovial proliferation; hematoxylin and eosin (H&E) stain

**Figure 9:**
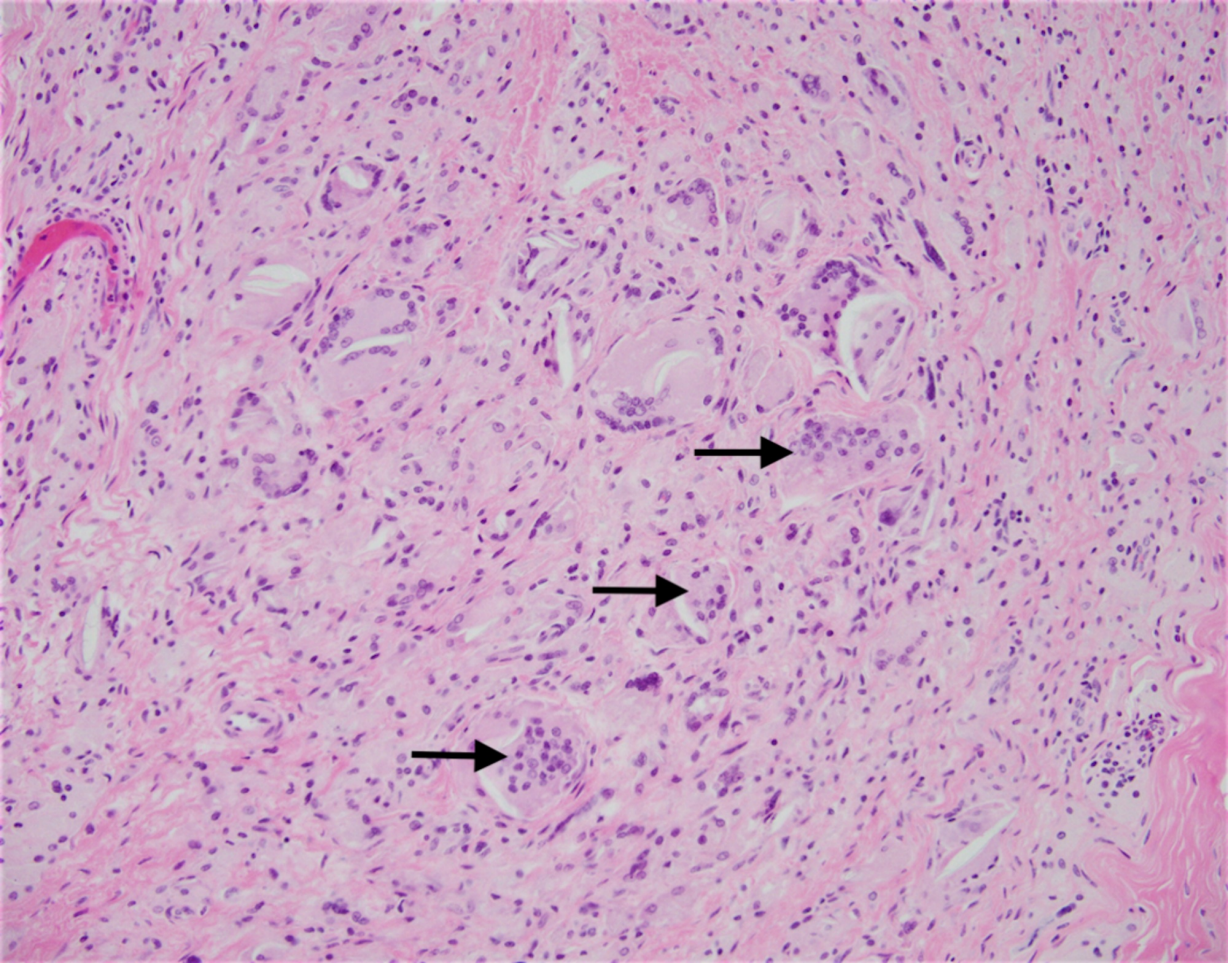
High power photomicrograph showing multiple foreign body giant cells in response to polarizable foreign material; hematoxylin and eosin (H&E) stain

**Figure 10:**
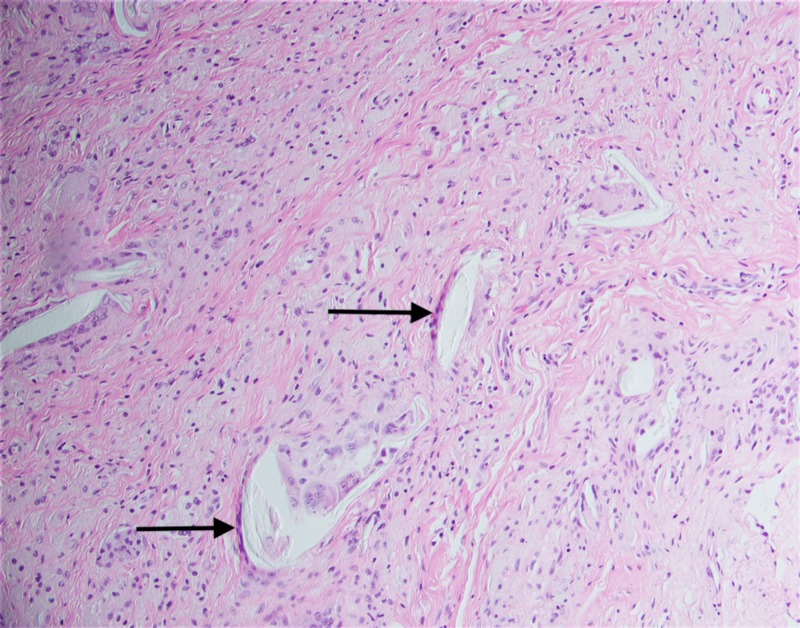
A photomicrograph showing multiple polarizable foreign material being engulfed by giant cells; hematoxylin and eosin (H&E) stain

A classical anterior approach to the knee was made through the patient’s previous scar. A medial parapatellar arthrotomy was performed, after which the knee joint was exposed, revealing extensive osteophytes around the patella (Figure 5). The osteophytes were removed and a medial release was performed, allowing for removal of the previous components (Figure 6). The femoral component was found to have bone ingrowth, which had encased the patella (Figure 7). Visualization of the bone-cement interface intra-operatively proved difficult. Upon gross visual inspection, it appeared that local long-term reaction at bone-cement interface had engulfed the cement and resulted in direct ingrowth of bone to implant. Bone-implant interface tissue was taken for histology examination. Microscopically, the sections examined showed papillary synovial proliferation which is consistent with the patient’s history of long-standing osteoarthritis (Figure 8). Multiple foreign body giant cells, which are formed by fused macrophages, are seen in response to polarizable foreign material (Figure 9). Orthopaedic implants can cause chronic inflammation and giant cell foreign body response as seen in this case (Figure 10).

After component removal, joint preparation was done in standard fashion by membranous tissue removal and minimal freshening of previous bone cuts. Then revision of knee components was performed as per standard technique.

**Figure 11:**
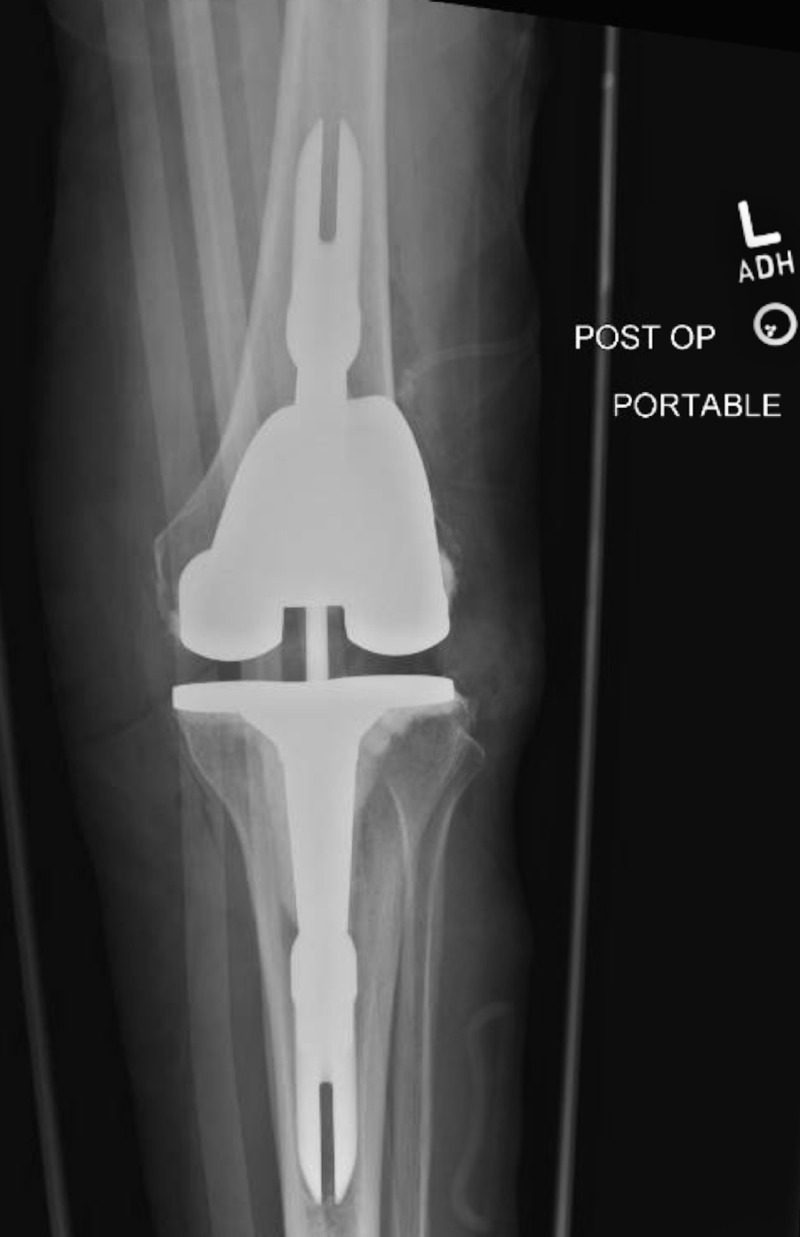
Postoperative anteroposterior radiograph confirming proper placement of left revision total knee arthroplasty (TKA) components

**Figure 12:**
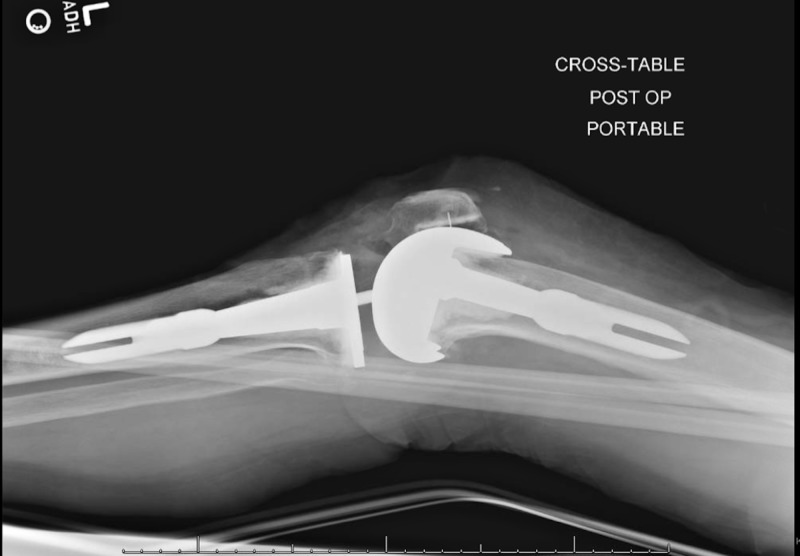
Postoperative lateral radiograph confirming proper placement of left revision total knee arthroplasty (TKA) components

**Figure 13:**
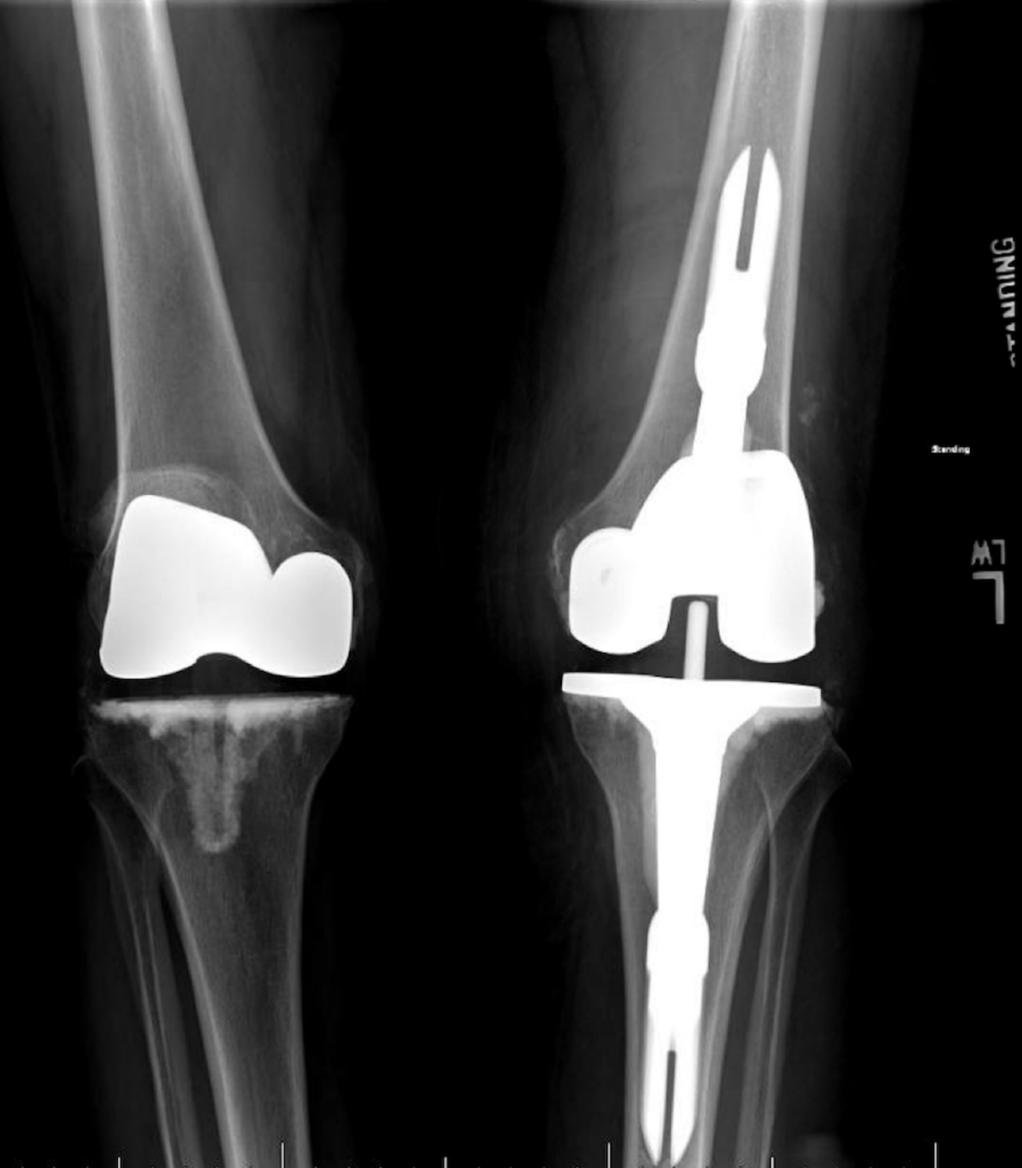
Postoperative radiograph at two-week follow up

**Figure 14:**
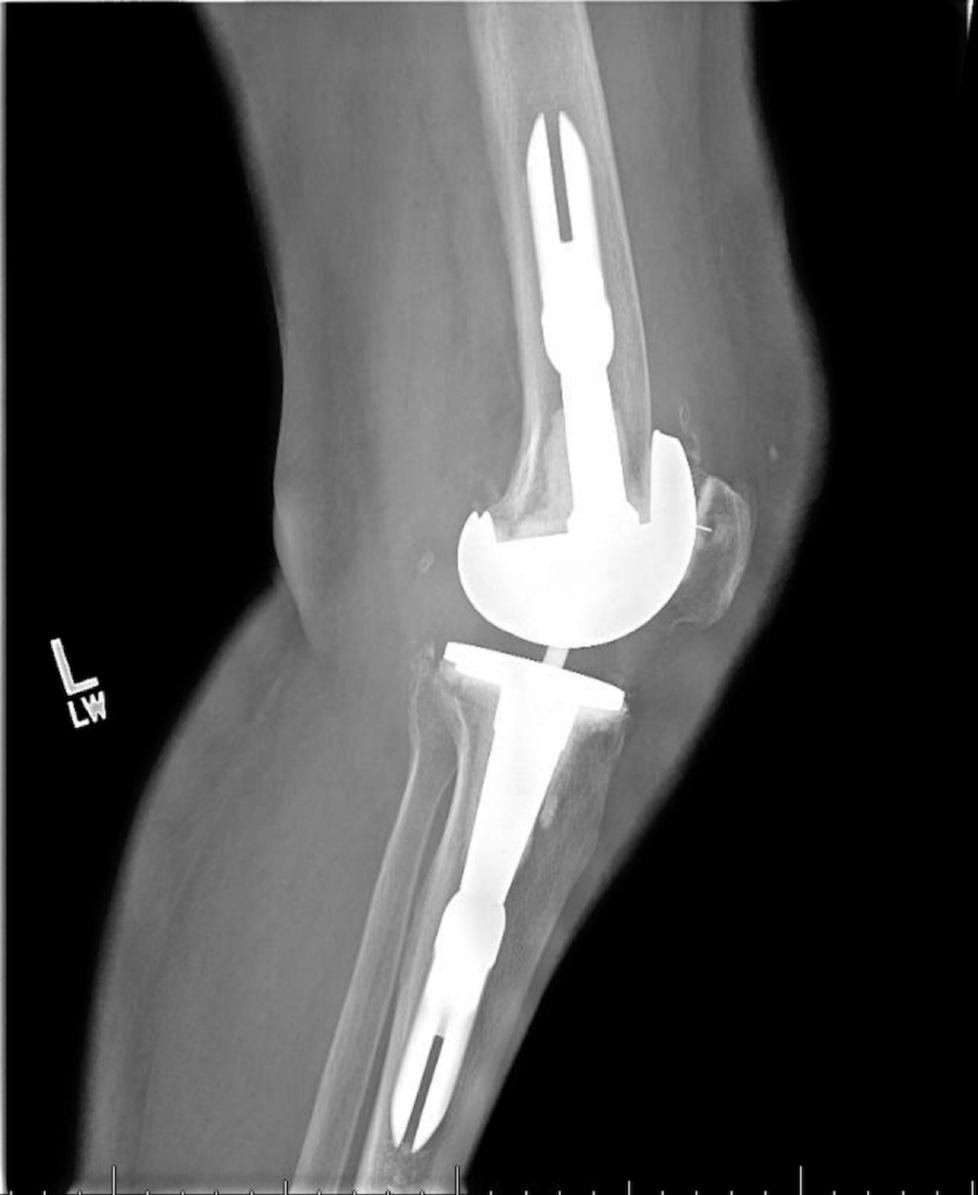
Postoperative radiograph at two-week follow up

Postoperative X-rays confirmed excellent placement of a left knee arthroplasty (Figures 11-12). The patient experienced no postoperative complications and was discharged from the hospital on postoperative day 2. She then followed up in the clinic two weeks postoperatively. The patient stated her pain was well controlled and had been working well with physical therapy. X-rays performed at this time reported that the left knee arthroplasty was in expected position with no evidence of hardware failure or loosening (Figures 13-14). She reported that she was pleased with her new prosthesis.

## Discussion

Several indications for revision TKA have been defined in literature including mechanical wear, aseptic loosening, infection, instability, osteolysis, pain, stiffness, malalignments, patellar complication, and periprosthetic fractures [7-8]. The most common indication for TKA revision within two years of surgery is infection [9]. However, implant instability is the leading overall cause for the need of revision surgery possibly due to the increasing probability of implant instability in the aging implant, as well as the decreasing age of patients receiving primary TKA [10-12]. From 1991-2011, 30% of primary TKAs were performed in patients aged less than 65 years [12]. This younger, more active patient population is at an increased risk for wear-induced osteolysis [13]. These patients also have an increased period for immune system mediated reactions to occur, potentially loosening the prosthesis [14].

Foreign body giant cell (FBGC) reaction is not commonly observed or described in knee arthroplasty. Giant cells are formed by the fusion of cells from the monocyte/macrophage lineage [6,15]. Cells of this lineage are among the first cells to come in contact with the implanted biomaterials [16]. These FBGCs are most commonly observed at the interface of tissue and implanted material, such as knee arthroplasty prosthesis in this case [6]. The purpose of these giant cells mirrors that of osteoclasts: degradation and resorption of the underlying substrate. The main difference between these two cell types is that osteoclasts adhere to the bone while FBGCs adhere primarily to synthetic surfaces [6].

Sheikh et al. examined osseointegration, the term given to the foreign body responses to implant surfaces [15]. This osseointegration, a direct result of the foreign body reaction, may allow for bone to grow over the implant surface given the right conditions. This process, while commonly identified in dental implants, has been rarely noted in the context of knee arthroplasty. Our histology reports suggest that the same kind of reaction also occurred in this patient, leading to revision knee arthroplasty. Harkel et al. demonstrated that multinucleated giant cells (MNGC) are unable to resorb bone with the same capabilities of osteoclasts [17]. However, the ability of MNGC’s to reabsorb hydroxyapatite, as well as to enhance osteoclast ability by more than 40-fold, was demonstrated.

The exact mechanism of this reaction of FBGCs and osteoclasts on bone, prostheses, and hydroxyapatite cement is incompletely understood [6]. Cytokine profiles in macrophages and FBGC’s in arthroplasty tissue are similar to those of mature osteoclasts [14]. However, what determines the differences in the course and progression of these reactions are unknown [18]. Although the mechanisms behind this reaction are incompletely understood, periodic radiological follow up may identify osteogenesis and osteolysis occurring around the arthroplasty. Further studies would contribute to a greater understanding of this pathological process.

AAHKS recommendation for clinic radiological follow up is annually for the first five years, biennial for years 6-10, and then either annual or biennial for the years thereafter [3]. However, the recommendations varied according to physician group sizes, patient population sizes, and the total number of surgeries performed, and adherence to guidelines in clinical practice is far from universal. De Pablo et al. reported that less than half of patients who received a total hip arthroplasty had consistent radiographic follow-up [19]. 

Joshi et al. examined a large number of joint arthroplasty patients who were lost to follow up and perceived that these patients had no issues with their new joint [20]. This study implied that patients who experience issues and require revision present to their surgeon with symptoms leading towards that diagnosis. However, in some patients, by the time symptoms arise, there may be an extensive destruction of periprosthetic bone [12]. Clinical symptoms may be preceded by radiologic signs of implant failure [12]. Early detection of implant failure by routine follow up may prevent the need for complex revision surgery, which is more costly and laborious process to the patient.

## Conclusions

FBGC and osteoclastic activity contributing to loosening is often a cause for component loosening in joint replacement surgeries. The fact that this patient remained clinically asymptomatic for over 15 years, until the aseptic osteolysis and component loosening became severe, highlights the importance of long-term follow up. With an increasingly younger patient age at the time of joint arthroplasty, long-term follow up to assess for signs and symptoms of implant failure is crucial for early diagnosis and to avoid more complex revision surgeries.
